# Efficacy of Location-Based Features for Survival Prediction of Patients With Glioblastoma Depending on Resection Status

**DOI:** 10.3389/fonc.2021.661123

**Published:** 2021-07-06

**Authors:** Madjid Soltani, Armin Bonakdar, Nastaran Shakourifar, Reza Babaei, Kaamran Raahemifar

**Affiliations:** ^1^Department of Mechanical Engineering, K. N. Toosi University of Technology, Tehran, Iran; ^2^Department of Electrical and Computer Engineering, University of Waterloo, Waterloo, ON, Canada; ^3^Centre for Biotechnology and Bioengineering (CBB), University of Waterloo, Waterloo, ON, Canada; ^4^Advanced Bioengineering Initiative Center, Computational Medicine Center, K. N. Toosi University of Technology, Tehran, Iran; ^5^College of Information Sciences and Technology (IST), Data Science and Artificial Intelligence Program, Penn State University, State College, Pennsylvania, PA, United States; ^6^Chemical Engineering Department, University of Waterloo, Waterloo, ON, Canada; ^7^Optometry & Vision Science Department, University of Waterloo, Waterloo, ON, Canada

**Keywords:** tumor location, feature selection, radiomics, glioblastoma, BraTS2019, artificial neural network, machine learning

## Abstract

Cancer stands out as one of the fatal diseases people are facing all the time. Each year, a countless number of people die because of the late diagnosis of cancer or wrong treatments. Glioma, one of the most common primary brain tumors, has different aggressiveness and sub-regions, which can affect the risk of disease. Although prediction of overall survival based on multimodal magnetic resonance imaging (MRI) is challenging, in this study, we assess if and how location-based features of tumors can affect overall survival prediction. This approach is evaluated independently and in combination with radiomic features. The process is carried out on a data set entailing MRI images of patients with glioblastoma. To assess the impact of resection status, the data set is divided into two groups, patients were reported as gross total resection and unknown resection status. Then, different machine learning algorithms were used to evaluate how location features are linked with overall survival. Results from regression models indicate that location-based features have considerable effects on the patients’ overall survival independently. Additionally, classifier models show an improvement in prediction accuracy by the addition of location-based features to radiomic features.

## Introduction

Glioma presents as a common intra-axial brain tumor. About one third of all brain tumors are gliomas (1), which begin in glial cells that support and protect neurons in the brain, like astrocytes, oligodendrocytes, and ependymal cells (2). It is categorized into two groups: low-grade glioma (grade I and II) and high-grade glioma (grade III and IV). Glioblastoma (GBM) is the most aggressive type among glioma tumors, with a 5-year survival rate of 5% (3).

Traditionally, pre-surgical overall survival (OS) was predicted by numerous factors, such as patient’s age, histopathological types, physical status, neurological disability, and medical image analysis. Medical image analysis and histopathological report are used to administer the cancer therapy. However, as the cancers’ variety and aggression are growing substantially, the tumor’s molecular analysis would be a great way to have a more accurate outlook on diagnosis and treatment. That is why the recent molecular pathological studies have shown significantly different OS for the higher-grade glioma patients with the same tumor histopathology (4). These findings indicate that the traditional survival prediction based on simple clinical information may not be adequately accurate. Besides, oncologists, as erroneous human beings, cannot consider all features in medical images. Instead, although automatic analysis of these tumors is challenging, it is a good alternative for traditional approaches and provides better accuracy.

Radiomics is an emerging method that can extract a large number of quantitative features from multidimensional medical images by using an automatic feature extraction algorithm. These features have the potential to give accurate characteristics that would not be considered by the naked eye. Radiomics leads to advanced image-based tumor phenotyping that provides valuable clinical information for OS prediction.

In this study, a data set consisting of multimodal MRI scans of brain tumor patients is used. Each of the sequences can show a specific part of the tumor brighter. The tumor segmentation map, which is essential for radiomic feature extraction, will be obtained by employing all of the sequences. These sequences include T1-weighted MRI (T1), T1-weighted MRI with contrast enhancement (T1CE), T2-weighted MRI (T2), fluid-attenuated inversion recovery (FLAIR), and so on.

The differences between the MRI acquisitions are two main parameters: repetition time (TR) and time to echo (TE). The time between successive pulse sequences applied to the same slice is called TR, whereas TE is the time between the delivery and reception of the radiofrequency (RF) pulse and the echo signal, respectively.

T1-weighted images are produced using short TR and TE. They are the most commonly used sequences for brain tumor structure analysis because they allow for easy annotation of the healthy tissues. In T1CE sequence images, the brain tumor borders are brighter because of the accumulation of contrast agents, and the necrotic core can be distinguished easily. Moreover, T2-weighted images are produced using long TR and TE and can show the edema region brighter than other parts. Since very long TR and TE are used in the imaging process of FLAIR, FLAIR is regarded as a highly effective sequence image to help distinguish the edema region from the cerebrospinal fluid (CSF). Incorporating these MRI sequences with different specifications, radiomic features are extracted from the images (5).

A review of radiomic-based techniques for quantitative imaging was investigated by Zou et al. (6). Also, various researches have been carried out in the last 5 years to evaluate the effectiveness of radiomic features and optimize their application (7) and (8). Prateek Prasanna (7) used computerized texture (i.e., radiomic) analysis to evaluate the efficacy of the peritumoral brain zone (PBZ) features from pre-operative MRI in predicting long- (>18 months) versus short-term (<7 months) survival in GBM, whereas Cho (8) applied radiomics to distinguish between high-grade and low-grade glioma and the efficacy of PBZ features from pre-operative MRI. Similarly, the research done by Weninger et al. (9) worked on “age-only regression model” and with the accuracy of 56% showing that adding radiomics to the age parameter does not necessarily improve the prediction accuracy for different resection statuses. Shboul et al. (10) used random forest regression (RFR) with radiomic features and Feng et al. (11) used linear models with geometrics. They achieved 58% and 62% accuracy, respectively (12). Other researches also evaluated radiomic features by using deep learning radiomics algorithm for gliomas (DRAG) (13), full-resolution residual convolutional neural network (FRRN) (14), and RFR with atlas locations, tumor’s relative size with “pseudo-3D” method (15), and RFR method (16).

Using the radiomic feature extraction method, a great number of features will be extracted. These features need to be analyzed by a proper model investigating the pattern between these features and the OS of a patient. Therefore, machine learning (ML) algorithms are chosen to analyze features.

Although some related factors have been clearly linked to the OS of patients with GBM, the impact of location on survival has been assessed less clearly. Motivated by this deficiency, this study aims to extract location-based features of GBM and evaluate how they affect the OS prediction independently and next to the radiomic features.

In this paper, we analyze the efficacy of the tumor’s location on BraTS 2019 data. It is important to note that to avoid imperfect segmentations affecting our predictions, we only use the training data sets of the BraTS 2019. The ground truth segmentations of the training data set were extracted by one to four experts with the same annotation protocol and also have been approved by experienced neuro-radiologists. To assess the effect of resection status, we divide the data set regarding patients’ resection status. Thus, patients with gross total resection status (GTR) and unavailable resection status (NA) are used independently.

Dividing the data set into two main subgroups, we first extract location-based features from MRI images. In this step, we use several regression algorithms to assess each feature’s influence on OS independently. Second, we extract radiomic features and implement feature reduction algorithms to remove redundant features. Finally, we concatenate radiomics and the location-based features, put them into multivariate prediction models, and evaluate the efficacy of adding the location-based features to radiomics on OS prediction.

## Materials

### Dataset

The BraTS challenge is held every year to compare different segmentation algorithms since 2012. Since 2017, quantitative image features have been investigated to see whether they can enrich the clinical insight and improve the prediction accuracy of the patients’ survival days (17).

In this project, BraTs 2019 glioma training data set (18–20) is used, which includes data from 211 GBM patients with OS ranging from 3 to 1,767 days. For each patient, four MRI acquisitions (T1, T1CE, T2, and T2-FLAIR), segmentation map including edema (ED), enhancing tumor (ET), and non-enhancing necrotic tumor core (NEC), the survival days, and the resection status are available.

The data set has been subdivided by resection status into patients reported as GTR, subtotal resection (STR), and NA. In BraTS 2018, a few patients were given as STR, but in BraTS 2019 data set, only data for patients reported as GTR and NA are available. These resection status differences can affect the training accuracy, so we chose to separately train the prediction models on GTR and NA resection statuses.

In the BraTS challenge, the patients’ OS has been categorized as long survivors (e.g., >15 months), short survivors (e.g., <10 months), and mid-survivors (e.g., between 10 and 15 months). We considered the long survival parameter (450 days) as the midpoint as we intended to have binary target values in our classification. In regression models, however, all the data were fitted to the exact survival days.

### Cohort Study

The BraTS data set samples are mostly from The Center for Biomedical Image Computing and Analytics from the University of Pennsylvania (CBICA) and The Cancer Imaging Archive (TCIA). Differences in population, imaging protocols, and treatment can affect the prediction models noticeably. The following paragraph will clarify the similarities of the data set provided.

**Figure 1:**
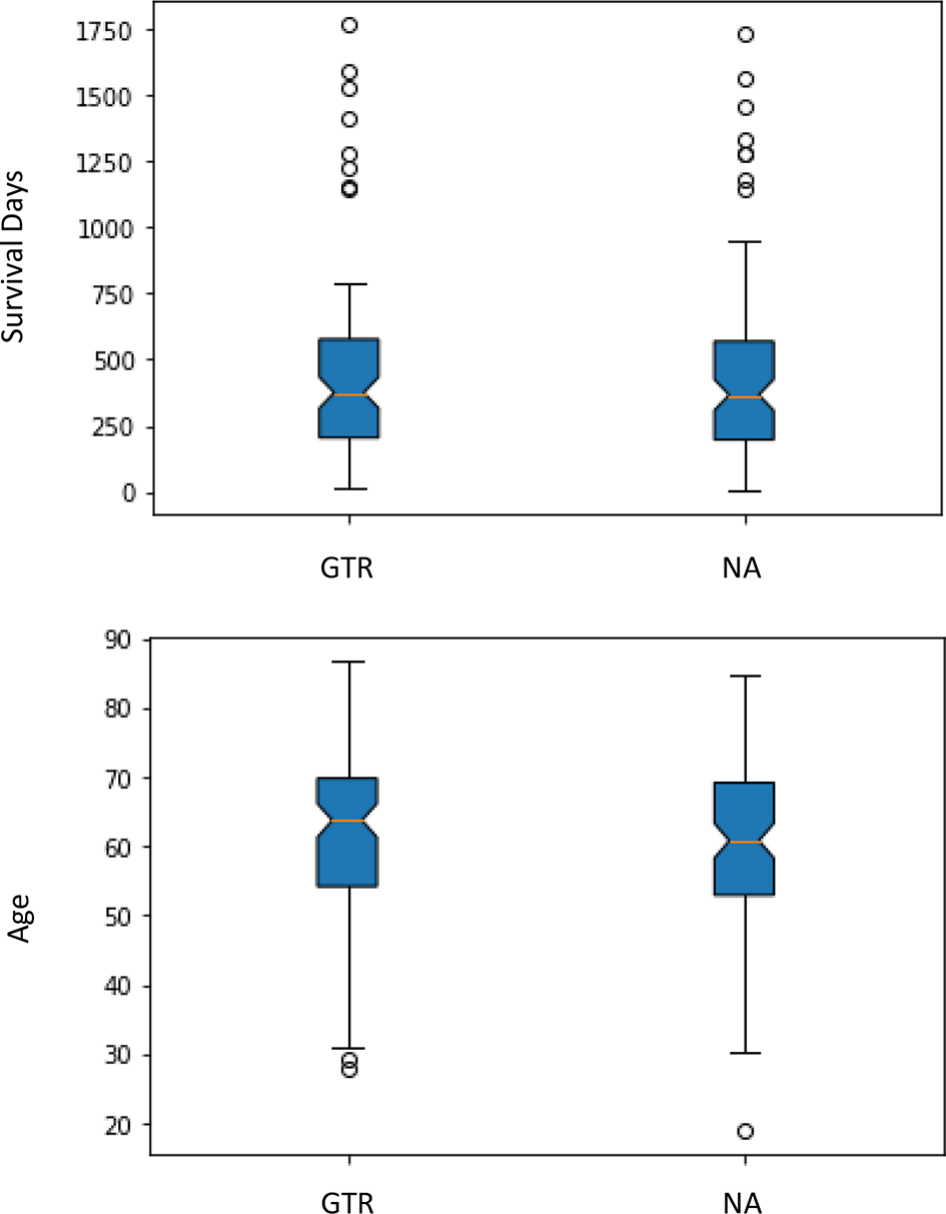
Differences in age and survival by resection status.

First, all the patients with GTR are from the CBICA institution, and patients with NA are from TCIA. Second, the one-way analysis of variance (ANOVA: *p*-value) is a parameter helping us to know whether the groups have significant statistical differences or not. In this data set, since the *p*-value is over 0.05, there are no significant statistical differences between age or survival for the different data sources and the different types of resection status, so it is rather safe to consider the groups with a similar statistical situation (**Figure 1**).

## Methods

**Figure 2:**
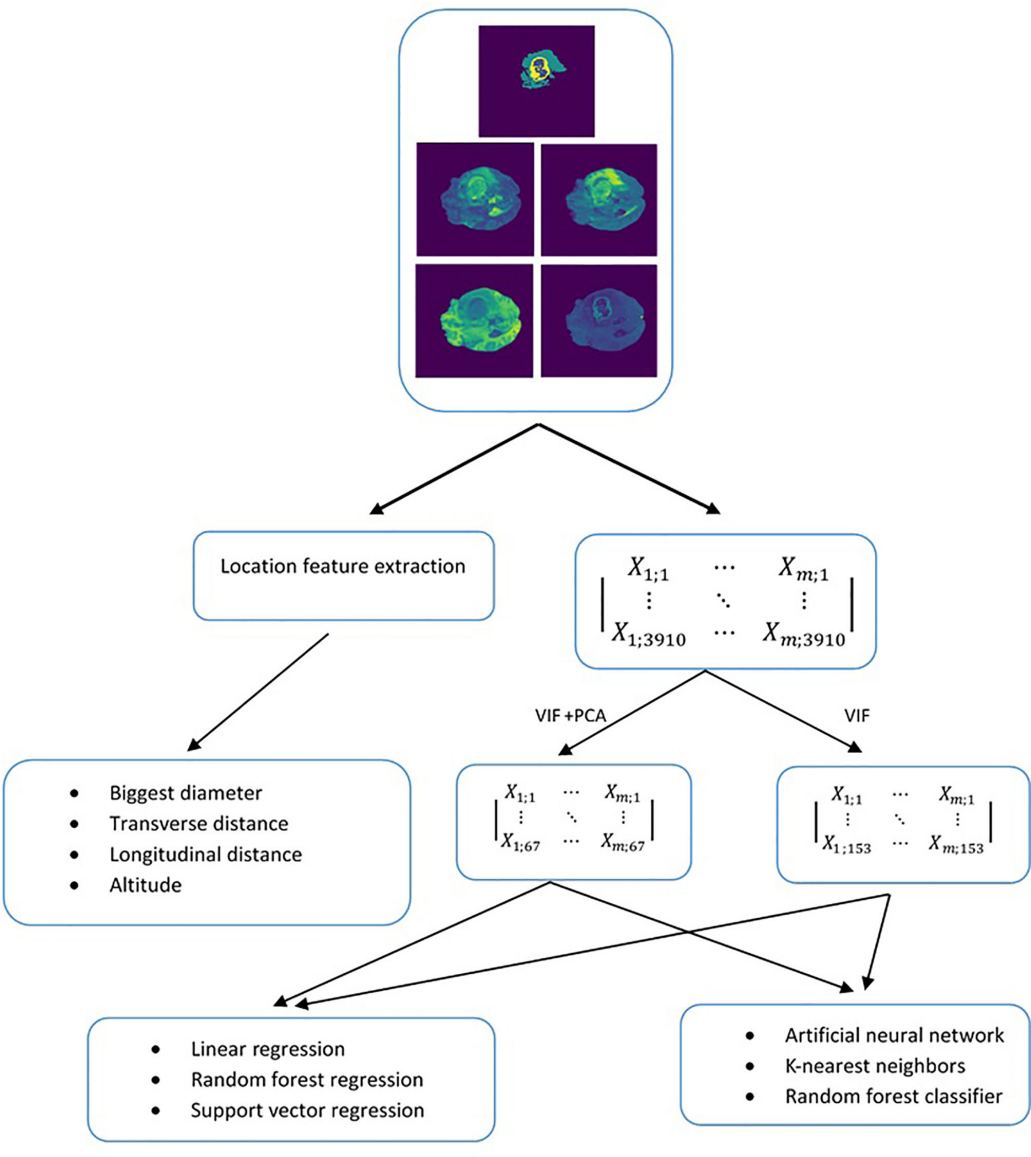
Methodology used to evaluate the predictiveness of location-based features independently and in combination with radiomics for overall survival.

The OS prediction model entails five main steps: (1) image preprocessing, (2) location features extraction, (3) radiomic features extraction, (4) unsupervised feature reduction, and (5) regression and classification predictive modeling. These major steps can be vividly seen in **Figure 2**.

### Image Preprocessing

MRI images have been acquired from different scanners, as well as various imaging protocols. Such differences undoubtedly lead to image intensities’ diversity, and subsequently, our features to be extracted will be affected. To tackle this problem, two different filters, N4 bias field correction (21) and z-score normalization, were used to remove local differences in image intensities and normalize the image to unit variance and zero mean, respectively.

1) Z-score normalization

The formula for calculating a z-score is:

Eq. (1) $$Z=\frac{x-\mu }{\sigma }$$

In Eq. 1, x, μ, and σ are the raw value, the mean value, and the standard deviation of pixels of the image, respectively. This procedure helped us to normalize all the images of the data set. In the end, the mean value of all pixels would be zero, and the standard deviation would be equal to one for all images.

2) N4 bias field correction

N4 bias field correction is a popular method in medical image preprocessing that uses a multi-scale optimization approach to correct low-frequency intensity non-uniformity present in MRI image data. In addition to the “real” pixel, the “MaskImage” can be used to specify the pixels required and avoid excessive processing.

### Radiomic Feature Extraction

In this step, radiomic features were extracted by implementing the *Pyradiomics* module (22). Generally, the extracted features are as follows:

- First Order Statistics
- Shape-based (3D)
- Shape-based (2D)
- Gray Level Co-occurrence Matrix
- Gray Level Run Length Matrix
- Gray Level Size Zone Matrix
- Neighboring Gray Tone Difference Matrix
- Gray Level Dependence Matrix

The number of extracted features from images was 3,910. It is clear from the data set supplied that the number of features for each patient is highly bigger than the number of patients. Such data are called wide data (23). Using such wide data usually results in overfitting in the training set. Consequently, to avoid overfitting, features have to be reduced.

### Standardization and Preselection of Features

First, as the scale of radiomic features varies significantly, we standardized them using the *scikit-learn* object *StandardScaler* to avoid their excessive influence on prediction models. Additionally, reducing radiomic features is compulsory because of their redundancy (24). So, different reduction methods were used to decrease radiomic features. In this study, supervised methods were implanted for patients with different resection status (the number of patients as GTR = 101, the number of patients as NA = 99), which are as follows:

#### Correlation Matrix

In this method, each feature was linearly regressed against other features. The correlation matrix is one of the simplest methods by which the pairwise correlations between single features can be investigated, and the representative features can be chosen (25). Next, a correlation matrix was made, and areas of high correlation (> 0.95) were reduced to the element with the highest variability.

#### Variance Inflation Factor (VIF)

To identify and exterminate multicollinearity, the VIF preselection method was implemented in the remaining features. A threshold of 10 is the recommended reference for this method. Consequently, a maximum VIF of 10 was chosen, and those with higher VIF factors were omitted.

#### Principal Component Analysis (PCA)

Next to VIF feature reduction, PCA was carried out on radiomic features to extract the vital information from the data set (26) and reduce the dimension (27) to avoid overfitting in learning algorithms. In this step, the features, which include 95% of the variance in the data, were kept.

#### Statistical Hypothesis Testing

Hypothesis tests are needed in this step to control the false discovery rate and reveal if single features have a considerable effect on OS prediction. The subset selected by VIF and PCA feature selection was tested with the Benjamini–Hochberg procedure (28), which controls the false discovery rate at a specific level α = 0.05.

### Location Feature Extraction

**Figure 3:**
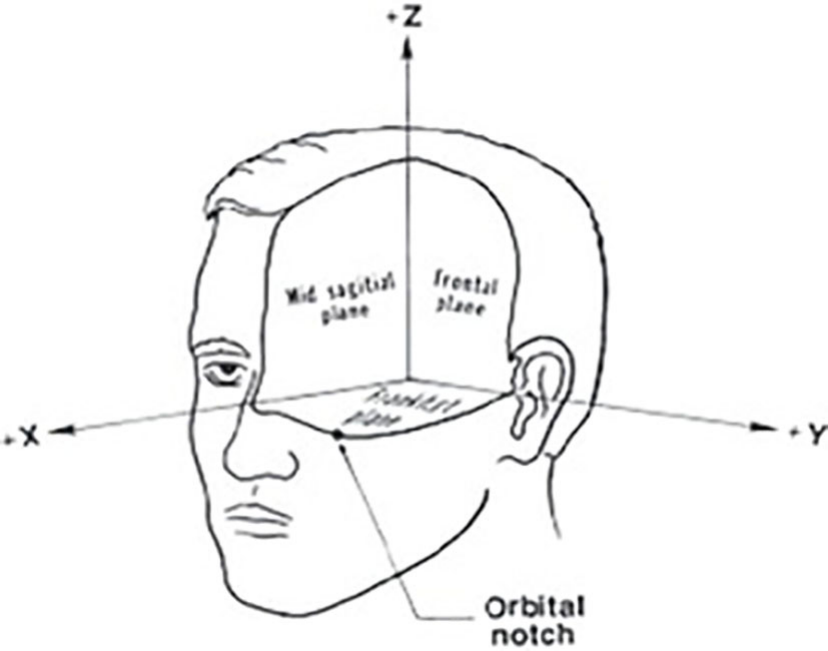
Cartesian coordinates system fitted on brain schematic (29).

In this project, the main concern goes to the tumor’s exact location and its diameter on patient survival rate. To find the tumor center’s location, first, the brain schematic was fitted in the Cartesian coordinates system (**Figure 3**). Then the exact tumor location was calculated using the following steps.

**Figure 4:**
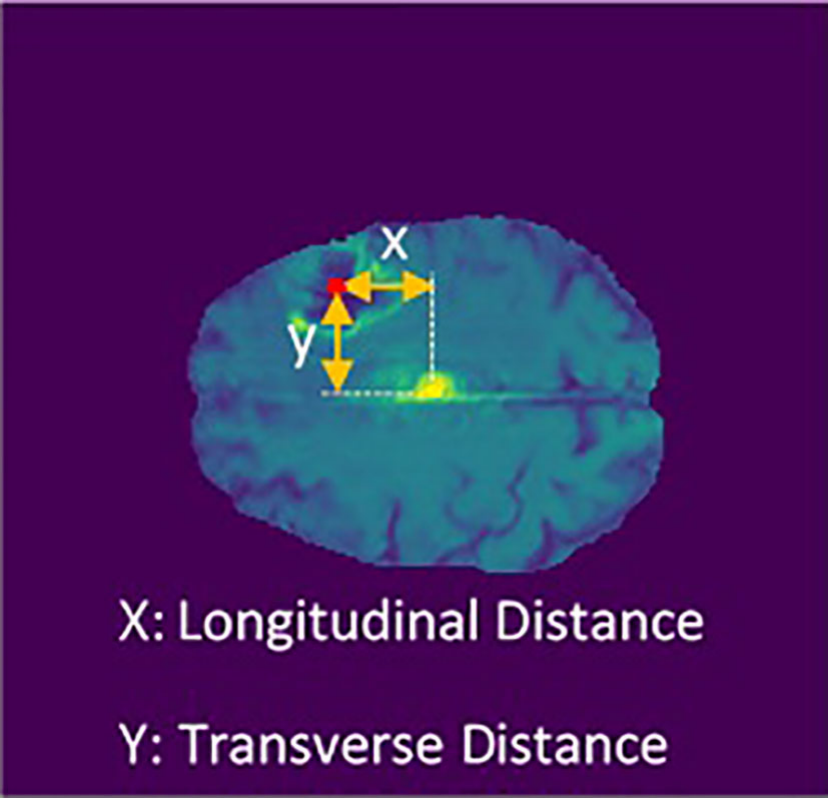
Longitudinal and transverse distance calculation.

Using the Euclidean distance measurement algorithm, the tumor’s transverse diameter was calculated in all the 154 transverse sections. Next, the section with the biggest diameter was considered as the tumor’s middle surface (Slice). The center of the tumor’s longitudinal (X) and transverse (Y) distance from the center of the brain (midline crossing) were calculated (**Figure 4**). Finally, the slice number, the diameter of the tumor, X, and Y were used in univariate and multivariate prediction models to evaluate their robustness. Implementing these steps helped to have clear information about the location of the tumor in the brain.

### Prediction Models

This project evaluates regression and classification predictive models simultaneously for patients with GTR and NA resection status. In regression predictive models, features were directly fitted to the patients’ survival days, whereas in classification, survival days were divided into two main groups (short and medium survival, < 450 days; long survival, > 450 days).

For univariate feature evaluation, linear regression (LR), random forest regression (RFR) models, and support vector regression (SVR) were used. LR attempts to model the linear relationship between two variables. One variable is considered an explanatory variable, and the other is regarded as a dependent variable. RFR fits several classifying decision trees on various sub-samples of the data set and uses averaging to improve the predictive accuracy and control over-fitting. SVR is considered a nonparametric technique as it relies on kernel functions. All regression models show the effect of location-based features independently.

For multivariate feature evaluation, we chose artificial neural network (ANN), random forest classifier (RFC), and k-nearest neighbors (KNN). An ANN is a computing system that operates like the human brain. It includes several interconnected nodes in different layers helping to find the complex correlation between the inputs and the target values. In the random forest, a large number of individual decision trees are used simultaneously to predict the output based on input values. Each tree in the random forest method leads to a prediction model, and the class with the most votes becomes our model’s prediction. KNN is one of the simplest ML supervised learning algorithms. KNN algorithm investigates the similarity between the new case and available cases and puts the new case into the most similar category. Consequently, all the available data are stored, and a new data point is made.

## Results

### Radiomic Feature Reduction Approaches

First, the correlation matrix clustering method reduced radiomic features from 3,910 to 1,601 based on the features’ pairwise correlation. Second, the VIF-based feature reduction algorithm removed redundant features with multicollinearity correlation and decreased the preselected features to 153. These extracted features were used in prediction models, as their number is acceptable for our limited data set. Furthermore, the PCA feature extraction method was used on VIF preselected features and reduced from 153 to 67. This allows us to see whether implementing different feature reduction methods at the same time can be effective in the prediction models or not.

### Hypothesis Testing

In the subset selected by VIF and PCA methods, hypothesis testing is feasible. After Benjamini–Hochberg correction on VIF-based and PCA-based subsets, only six and four radiomic features remained significant, respectively.

### Univariate Prediction Models

**Figure 5:**
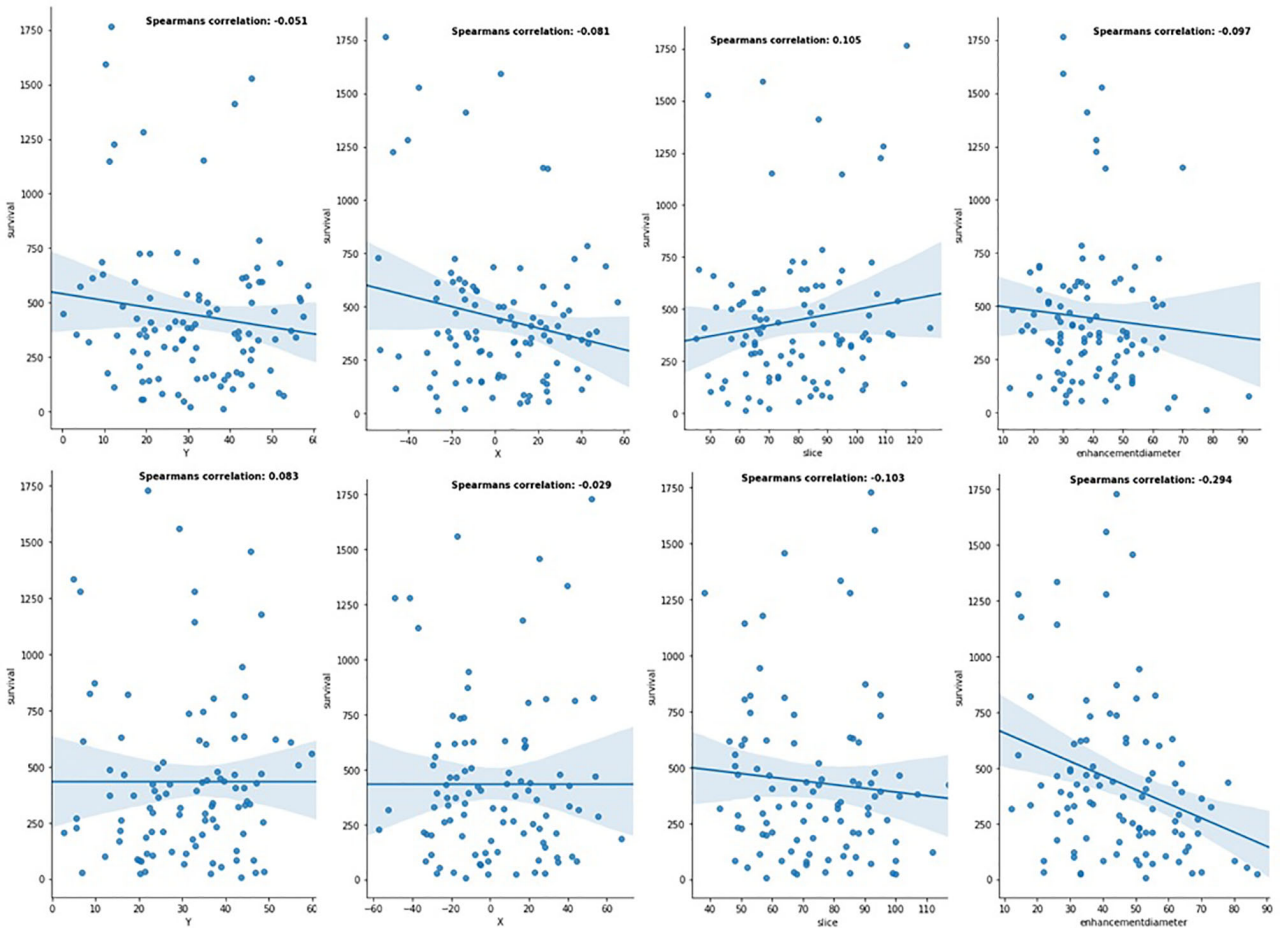
Linear relationship between location-based features and patients' OS in the different resection status (the first row is for patients with GTR resection status, and the second row is for patients with NA resection status).

First, the robustness of location-based features was investigated independently. Results from LR (**Figure 5**) indicated that X, Y, slice (tumor’s height from the bottom of the brain), and tumor diameter features had impact on prediction of patients with GTR. However, the results for patients with NA were not promising. This shows the effect of resection status on better evaluation. Also, using RFR and SVR, Spearman R, mean square error (MES), median absolute error (MAE), mean absolute error, and *p*-value were calculated. Similarly, they indicated better influences on GTR patients (**Table 1**).

**Table 1:** Performance comparison of regression models for the different types of resection status.

Feature	Model	Spearman R	MSE	Median AE	Mean AE	p value	Model	Spearman R	MSE	Median AE	Mean AE	p value
	NA resection status						GTR resection status					
**Diameter**	**LR**	-0.294	78049.28	223.23	239.85	0.82	**LR**	-0.097	37428.03	159.01	168.15	0.36
	**RFR**	-0.06	159280.9	282.93	331.82	0.85	**RFR**	-0.41	58338.01	131.43	187.91	0.22
	**SVR**	0.05	81374.4	126.03	190.16	0.88	**SVR**	-0.21	26817.23	133.65	138.92	0.55
**X**	** **						** **					
	**LR**	-0.03	80363.53	122.71	206.21	0.08	**LR**	-0.081	42009.9	139.48	154.29	0.90
	**RFR**	-0.39	219679.9	315.4	369.96	0.25	**RFR**	-0.23	56161.69	175.35	184.85	0.51
	**SVR**	-0.1	81958.81	126.64	190.72	0.77	**SVR**	0.13	26420.36	133.99	139.04	0.70
**Y**	** **						** **					
	**LR**	0.083	77261.72	125.21	203.08	0.75	**LR**	-0.51	38447.94	174.54	165.92	0.005
	**RFR**	0.6	78563.14	180.54	219.7	0.06	**RFR**	-0.41	104772.9	229.62	268.94	0.23
	**SVR**	0.11	81732.79	125.23	190.29	0.75	**SVR**	-0.71	26733.7	132.54	139.14	0.01
**Slice**	** **						** **					
	**LR**	-0.103	74304.09	151.36	203.15	0.60	**LR**	0.105	39080.92	161.89	167.2	0.04
	**RFR**	-0.16	141523.20	329.26	333.14	0.65	**RFR**	-0.13	36495.76	159.84	162.59	0.04
	**SVR**	0.13	81892.96	128.79	189.75	0.70	**SVR**	-0.11	26869.68	134.1	139.53	0.03

Note that MSE, Median AE and Mean AE are Mean Square Error, Median Absolute Error and Mean Absolute Error respectively.

### Multivariate Prediction Models

All classifier models were used on patients reported as GTR and NA independently, and the results were provided in **Tables 2** and **3**. As the patients’ OS were divided into two classes (long-survivors > 450 days, short-survivors < 450 days), and there was no significant imbalance in the data set, the binary cross-entropy was used as the loss function of the algorithms. ANN, RFC, and KNN were used on two different radiomic feature reduction methods to assess the influence of adding the location-based features to radiomics. The following statistics were computed: accuracy, precision, sensitivity, and specificity. Furthermore, the results for the GTR data set are much better than NAs, which shows the importance of using univariate resection status in the prediction models. In the GTR data set, ANN had the highest accuracy of 69% in VIF-selected radiomic features. Furthermore, with the addition of location-based features, the accuracy of ANN rose by 9%, which is slightly higher than other classification methods. On the contrary, in the NA data set, the highest accuracies have been achieved from RFC with 60% for PCA-selected radiomic features. Also, adding the location-based features in the RFC method showed an increase of 6%.

**Table 2:** Performance comparison of classification models for patients’ reported as GTR.

Model	Accuracy	Precision	Sensitivity	Specificity	Model	Accuracy	Precision	Sensitivity	Specificity
**VIF-based feature subset**					**VIF-based feature subset and location-based features**				
ANN	0.69	0.5	0.77	0.5	ANN	0.78	0.6	0.8	0.6
KNN	0.57	0.66	0.4	0.77	KNN	0.63	0.71	0.5	0.77
RFC	0.68	0.83	0.5	0.88	RFC	0.73	0.85	0.6	0.88
**PCA-based feature subset**					**PCA-based feature subset and location-based features**				
ANN	0.62	0.55	0.75	0.42	ANN	0.66	0.6	0.8	0.6
KNN	0.63	0.66	0.6	0.66	KNN	0.63	0.71	0.5	0.77
RFC	0.68	0.7	0.7	0.66	RFC	0.68	0.75	0.6	0.77

**Table 3:** Performance comparison of classification models for patients’ reported as NA.

Model	Accuracy	Precision	Sensitivity	Specificity	Model	Accuracy	Precision	Sensitivity	Specificity
**VIF-based feature subset**					**VIF-based feature subset and location-based features**				
ANN	0.46	0.37	0.5	0.44	ANN	0.53	0.44	0.66	0.44
KNN	0.6	0.5	0.66	0.55	KNN	0.6	0.5	0.33	0.77
RFC	0.46	0.37	0.5	0.44	RFC	0.53	0.44	0.66	0.44
**PCA-based feature subset**					**PCA-based feature subset and location-based features**				
ANN	0.65	0.25	0.72	0.25	ANN	0.65	0.33	0.75	0.33
KNN	0.53	0.42	0.5	0.55	KNN	0.6	0.5	0.33	0.77
RFC	0.6	0.5	0.5	0.66	RFC	0.66	0.57	0.66	0.66

## Discussion

Previously published research worked on the applications of artificial intelligence (AI), especially convolutional neural networks (CNN). Although various methods were used to predict the patients’ OS, a few researchers divided the data set into subgroups based on patients’ resection status (9). Most researches were done irrespective of the patients’ resection status. Like the TCGA glioblastoma data set studied by Gutman et al. (30), the medical reports of patients with GB who have been diagnosed at the University of Pennsylvania investigated by Macyszyn et al. (31), researches done by Kickingereder et al. (32), Lao et al. (33), and Li et al. (34). Also, radiomics were used in the quantitative volumetric analysis of MRI (35) and 3D deep feature learning (4), without considering the patients’ resection status. The BraTS 2019 data set contains data of the patients with glioblastoma that underwent GTR and NA resection status. In this research, we separated them to evaluate the effectiveness of resection status. Results from regression and classification models indicated that the resection status is an important parameter in the prediction models. The models were far more easily trained on the data set with unique resection status (GTR) than the data set with various resection statuses (NA).

In this paper, binary output has been chosen for classification methods. This is the main reason, which resulted in higher accuracy compared to the research carried out by Weninger et al. (9), who categorized the OS into three subgroups. Additionally, results in the LR method investigating the effect of location-based features independently showed that these features can play an important role in patients’ OS. Although ML algorithms can identify the influence of location-based features, a larger data set is required to make this evaluation more applicable for GBM diagnosis. Many quantitative image analysis researches have been done based on radiomics. Sun et al. (36) used various ML algorithms on different radiomic feature groups and the maximum performance of feature selection and ML methods was 0.682. Wijethilake et al. (37) evaluated the influence of radiomics with different ML algorithms, and their results varied between 40% and 53%. Similarly, Baid et al. (38) used multi-layer perceptron (MLP) on radiomic features and the accuracy and *p* value of the algorithm were 0.571 and 0,427 respectively. Such results show that radiomic features are not promising enough in patients’ OS prediction. So, to increase the robustness of radiomics, they have to be combined with other effective features (38) in more powerful methods.

In the results, we indicated that the location-based features positively impact patients’ OS either independently or with radiomics. However, we do not consider our algorithms as the best strategies, and future studies, which use a larger data set and new learning strategies, can extract better results.

Cancer, especially GBM, is an ill-posed problem. A staggering number of parameters can be adequate for patients’ OS. However, current computer-aided methods for diagnosis and treatment have helped clinicians to analyze the patients’ disease and their treatment more quantitatively. That is why computational analysis of medical images has gained popularity in recent decades. There would be a great potential for researchers to use the power of AI on medical images.

Like many previous published papers, in this research, accuracies for both regression and classification models are not very high. This is no doubt because of the limited data set used. By using a larger data set, we can tackle the overfitting problem in the training and the validation set and extract higher precision and accuracy from learning algorithms to take advantage of algorithms in medical treatment.

## Conclusion

In this paper, the BraTs 2019 data set was used. The results showed that adding the brain tumor location feature to the extracted radiomic features can improve the accuracy of the prediction models. To demonstrate this, we first evaluated the influence of the location-based features independently with the regression models and then, we added them to the radiomics in the classification models. Results indicated their robustness even in this limited data set. Also, dividing the data set into two different groups based on patients’ resection status helped us to highlight the positive effect of the unique resection status on a more accurate prediction of patients’ OS.

To move from fundamental research to translational medicine, future studies have to be done on a larger data set, which includes more information about patients’ physical and social health. It seems that this information, along with quantitative features from patients’ medical images, can have an excellent potential for OS prediction.

## Data Availability Statement

Publicly available datasets were analyzed in this study. This data can be found here: https://www.med.upenn.edu/sbia/brats2018/registration.html.

## Ethics Statement

Ethical review and approval was not required for the study on human participants in accordance with the local legislation and institutional requirements. Written informed consent for participation was not required for this study in accordance with the national legislation and the institutional requirements.

## Author Contributions

AB, NS, and RB preprocessed the data set, performed algorithm implementation, and wrote the manuscript. MS and KR supervised the work, read and edited the manuscript, and discussed the results. All authors contributed to the article and approved the submitted version.

## Conflict of Interest

The authors declare that the research was conducted in the absence of any commercial or financial relationships that could be construed as a potential conflict of interest.

## References

[1] Atkinson M Juhász C Shah J Guo X Kupsky W Fuerst D. Paradoxical Imaging Findings in Cerebral Gliomas. J Neurological Sci (2008) 269:180–3. 10.1016/j.jns.2007.12.029 18255100

[2] Goodenberger ML Jenkins RB. Genetics of Adult Glioma. Cancer Genet (2012) 205:613–21. 10.1016/j.cancergen.2012.10.009 23238284

[3] Wang Y Zhao W Xiao Z Guan G Liu X Zhuang M. A Risk Signature With Four Autophagy-Related Genes for Predicting Survival of Glioblastoma Multiforme. J Cell Mol Med (2020) 24:3807–21. 10.1111/jcmm.14938 PMC7171404 32065482

[4] Nie D Lu J Zhang H Adeli E Wang J Yu Z. Multi-Channel 3D Deep Feature Learning for Survival Time Prediction of Brain Tumor Patients Using Multi-Modal Neuroimages. Sci Rep (2019) 9:1103. 10.1038/s41598-018-37387-9 30705340 PMC6355868

[5] Liu J Li M Wang J Wu F Liu T Pan Y. A Survey of MRI-Based Brain Tumor Segmentation Methods. Tsinghua Sci Technol (2014) 19:578–95. 10.1109/TST.2014.6961028

[6] Zhou M Scott J Chaudhury B Hall L Goldgof D Yeom KW. Radiomics in Brain Tumor: Image Assessment, Quantitative Feature Descriptors, and Machine-Learning Approaches. Am J Neuroradiol (2018) 39:208–16. 10.3174/ajnr.A5391 PMC5812810 28982791

[7] Prasanna P Patel J Partovi S Madabhushi A Tiwari P. Radiomic Features From the Peritumoral Brain Parenchyma on Treatment-Naïve Multi-Parametric MR Imaging Predict Long Versus Short-Term Survival in Glioblastoma Multiforme: Preliminary Findings. Eur Radiol (2017) 27:4188–97. 10.1007/s00330-016-4637-3 PMC5403632 27778090

[8] Cho H Lee S Kim J Park H. Classification of the Glioma Grading Using Radiomics Analysis. Peer J (2018) 6:e5982. 10.7717/peerj.5982 30498643 PMC6252243

[9] Weninger L Haarburger C Merhof D. Robustness of Radiomics for Survival Prediction of Brain Tumor Patients Depending on Resection Status. Front Comput Neurosci (2019) 13:73. 10.3389/fncom.2019.00073 31780915 PMC6857096

[10] Shboul ZA Vidyaratne L Alam M Iftekharuddin KM. Glioblastoma and Survival Prediction. Lecture Notes Comput Sci (2018) 10670:358–68. 10.1007/978-3-319-75238-9_31 PMC5999323 30016377

[11] Feng X Tustison N Patel S Meyer C. Brain Tumor Segmentation Using an Ensemble of 3D U-Nets and Overall Survival Prediction Using Radiomic Features. Front Comput Neurosci (2020) 14:25. 10.3389/fncom.2020.00025 32322196 PMC7158872

[12] Bakas S Reyes M Jakab A Bauer S Rempfler M Crimi A. Identifying the Best Machine Learning Algorithms for Brain Tumor Segmentation, Progression Assessment, and Overall Survival Prediction in the BRATS Challenge. (2018). 10.17863/CAM.38755

[13] Baid U Talbar S Rane S Gupta S Thakur M Moiyadi A. Deep Learning Radiomics Algorithm for Gliomas (DRAG) Model: A Novel Approach Using 3D UNET Based Deep Convolutional Neural Network for Predicting Survival in Gliomas. Lecture Notes Comput Sci (2019) 11384369–79. 10.1007/978-3-030-11726-9_33

[14] Jungo A McKinley R Meier R Knecht U Vera L Pérez-Beteta J. Towards Uncertainty-Assisted Brain Tumor Segmentation and Survival Prediction. Lecture Notes Comput Sci (2018) 11384:474–85. 10.1007/978-3-319-75238-9_40

[15] Puybareau E Tochon G Chazalon J Fabrizio J. Segmentation of Gliomas and Prediction of Patient Overall Survival: A Simple and Fast Procedure. Lecture Notes Comput Sci (2019) 11384:199–209. 10.1007/978-3-030-11726-9_18

[16] Sun L Zhang S Luo L. Tumor Segmentation and Survival Prediction in Glioma With Deep Learning. Lecture Notes Comput Sci (2019). 10.1007/978-3-030-11726-9_8 PMC6707136 31474816

[17] Menze BH Jakab A Bauer S Kalpathy-Cramer J Farahani K Kirby J. The Multimodal Brain Tumor Image Segmentation Benchmark (Brats). IEEE Trans Med Imaging (2015) 34(10):1993–2024. 10.1109/TMI.2014.2377694 25494501 PMC4833122

[18] Bakas S Akbari H Sotiras A Bilello M Rozycki M Kirby JS. Advancing the Cancer Genome Atlas Glioma MRI Collections With Expert Segmentation Labels and Radiomic Features. Nat Sci Data (2017) 4:170117. 10.1038/sdata.2017.117 PMC5685212 28872634

[19] Bakas S Akbari H Sotiras A Bilello M Rozycki M Kirby JS. Segmentation Labels and Radiomic Features for the Pre-Operative Scans of the TCGA-GBM Collection. Cancer Imaging Arch (2017). 10.7937/K9/TCIA.2017.KLXWJJ1Q PMC5685212 28872634

[20] Bakas S Akbari H Sotiras A Bilello M Rozycki M Kirby JS. Segmentation Labels and Radiomic Features for the Pre-Operative Scans of the TCGA-LGG Collection. Cancer Imaging Arch (2017). 10.7937/K9/TCIA.2017.GJQ7R0EF PMC5685212 28872634

[21] Tustison N Avants B Cook P Zheng Y Egan A Yushkevich P. N4itk: Improved N3 Bias Correction. IEEE Trans Med Imaging (2010) 29(6):1310–20. 10.1109/TMI.2010.2046908 PMC3071855 20378467

[22] Griethuysen J Fedorov A Parmar C Hosny A Aucoin N Narayan V. Computational Radiomics System to Decode the Radiographic Phenotype. J AACR (2017) 77(21):e104–7. 10.1158/0008-5472.CAN-17-0339 PMC5672828 29092951

[23] Bzdok D. Classical Statistics and Statistical Learning in Imaging Neuroscience. Front Neurosci (2017) 11:543. 10.3389/fnins.2017.00543 29056896 PMC5635056

[24] Rizzo S Botta F Raimondi S Origgi D Fanciullo C Giuseppe A. Radiomics: The Facts and the Challenges of Image Analysis. Eur Radiol Exp (2018) 2:36. 10.1186/s41747-018-0068-z 30426318 PMC6234198

[25] Gillies R Kinahan P Hricak H. Radiomics: Images Are More Than Pictures, They Are Data. RSNA J Radiol (2015) 278:563–77. 10.1148/radiol.2015151169 PMC4734157 26579733

[26] Abdi H Williams L. Principal Component Analysis. WIREs Comput Stat (2010) 2:433–59. 10.1002/wics.101

[27] Kambhatla N Leen T. Dimension Reduction by Local Principal Component Analysis. MIT Press J Neural Comput (2006) 9:1493–516. 10.1162/neco.1997.9.7.1493

[28] Benjamini Y Hochberg Y. Controlling the False Discovery Rate: A Practical and Powerful Approach to Multiple Testing. J R Stat Soc Ser B (1995) 57:289–300. 10.17863/CAM.38755

[29] Rash CE. Helmet-Mounted Displays: Design Issues for Rotary-Wing Aircraft. (2001). 10.1117/3.397108

[30] Gutman D Cooper L Hwang S Holder C Gao J Aurora T. MR Imaging Predictors of Molecular Profile and Survival: Multi-Institutional Study of the TCGA Glioblastoma Data Set. RSNA J Radiol (2013) 267:560–9. 10.1148/radiol.13120118 PMC3632807 23392431

[31] Macyszyn L Akbari H Pisapia J Da X Attiah M Pigrish V. Imaging Patterns Predict Patient Survival and Molecular Subtype in Glioblastoma via Machine Learning Techniques. J Neuro-Oncol (2015) 18:417–25. 10.1093/neuonc/nov127 PMC4767233 26188015

[32] Kickingereder P Burth S Wick A Götz M Eidel O Schlemmer H. Radiomic Profiling of Glioblastoma: Identifying an Imaging Predictor of Patient Survival With Improved Performance Over Established Clinical and Radiologic Risk Models. RSNA J Radiol (2016) 280:880–9. 10.1148/radiol.2016160845 27326665

[33] Lao J Chen Y Li Z Li Q Zhang J Liu J. A Deep Learning-Based Radiomics Model for Prediction of Survival in Glioblastoma Multiforme. Sci Rep (2017) 7:10353. 10.1038/s41598-017-10649-8 28871110 PMC5583361

[34] Li Q Bai H Chen Y Sun Q Liu L Zhou S. A Fully-Automatic Multiparametric Radiomics Model: Towards Reproducible and Prognostic Imaging Signature for Prediction of Overall Survival in Glioblastoma Multiforme. Sci Rep (2017) 7:14. 10.1038/s41598-017-14753-7 29085044 PMC5662697

[35] Zhang Z Jiang H Chen X Bai J Cui Y Ren X. Identifying the Survival Subtypes of Glioblastoma by Quantitative Volumetric Analysis of MRI. J Neurooncol (2014) 119:207–14. 10.1007/s11060-014-1478-2 24828264

[36] Sun W Jiang M Dang J Chang P Yin F. Effect of Machine Learning Methods on Predicting NSCLC Overall Survival Time Based on Radiomics Analysis. Radiat Oncol (2018) 13:197. 10.1186/s13014-018-1140-9 30290849 PMC6173915

[37] Wijethilake N Islam M Ren H. Radiogenomics Model for Overall Survival Prediction of Glioblastoma. Med Biol Eng Comput (2020) 58:1767–77. 10.1007/s11517-020-02179-9 32488372

[38] Baid U Rane S Talbar S Gupta S Thakur M Moiyadi A. Overall Survival Prediction in Glioblastoma With Radiomic Features Using Machine Learning. Front Comput Neurosci (2020) 14:61. 10.3389/fncom.2020.00061 32848682 PMC7417437

