# Erratum: Efficacy of Location-Based Features for Survival Prediction of Patients With Glioblastoma Depending on Resection Status

**DOI:** 10.3389/fonc.2021.745820

**Published:** 2021-08-05

**Authors:** Frontiers Production Office

**Affiliations:** Frontiers Media SA, Lausanne, Switzerland

**Keywords:** tumor location, feature selection, radiomics, glioblastoma, BraTS2019, artificial neural network, machine learning

Due to a production error, an author name was incorrectly spelled as ”Reza Babaie”. The correct spelling is ”Reza Babaei”. The publisher apologizes for this mistake.

The original version of this article has been updated.

